# Association between high HBV-DNA viral load and liver metastasis risk in patients with nasopharyngeal carcinoma

**DOI:** 10.1016/j.virusres.2025.199651

**Published:** 2025-10-25

**Authors:** Xiaofan Wang, Ying Bao, Sheng Yin, Yizhi Peng

**Affiliations:** a Department of Clinical Laboratory, Hunan Cancer Hospital/the Affiliated Cancer Hospital of Xiangya School of Medicine, Central South University, Changsha Province 410031, China; b Department of Clinical Laboratory, Taizhou Central Hospital (Taizhou University Hospital), Taizhou, Zhejiang Province 318000, China; c Department of Clinical Diagnosis, Laboratory of Beijing Tiantan Hospital and Capital Medical University, No.119, South Fourth Ring West Road, Fengtai District, Beijing 100070, China

**Keywords:** Nasopharyngeal carcinoma, Hepatic metastases, HBV-DNA

## Abstract

A total of 976 patients with nasopharyngeal carcinoma (NPC) confirmed by pathology in Xiangya Cancer Hospital of Central South University from May 2017 to December 2022 were recruited for the study. Meanwhile, the HBV-DNA was detected to explore the relationship between HBV-DNA and liver metastasis of NPC. The results indicated that there was no significant difference in the liver metastasis rate between the HBV-negative group and the HBV-positive group. The liver metastasis rate in the low HBV-DNA viral load group was lower than that in the high HBV-DNA viral load group (*p* = 0.049). Multivariate logistic regression analysis indicated that high HBV-DNA viral load was a risk factor for liver metastasis in HBV-positive NPC patients (OR=2.661, 95 %CI 1.32–4.39, *p* = 0.044). The HBV infection status itself had no significant association with whether NPC had liver metastasis. However, a high HBV-DNA viral load increased the risk of liver metastasis in patients with NPC.

## Highlights

• A high HBV-DNA viral load increased the risk of liver metastasis in patients with NPC. Active HBV replication may impair hepatic immune surveillance, promoting metastatic spread.
• Clinical vigilance and antiviral therapy are recommended for NPC patients with high HBV-DNA loads to mitigate liver metastasis risk.

## 1 Introduction

NPC was a malignant epithelial tumor originating from the nasopharyngeal mucosa, with significant and distinctive racial and geographical distribution characteristics (Luo, 2023; Wong et al., 2021). According to global cancer statistics for 2020, more than 75% of NPC cases occurred in Southeast Asia and Southern China (Sung et al., 2021). In China, non-keratinizing undifferentiated carcinoma was the most common pathological type of NPC (Stepan et al., 2021). Its rate of distant metastasis was higher than that of the other two types (squamous cell carcinoma and keratinizing undifferentiated carcinoma) (Stepan et al., 2021), with the primary sites of metastasis being the bones, lungs, liver, and distant lymph nodes (Tang et al., 2018). The liver was one of the most common metastatic sites for NPC and was usually multifocal, and was considered an independent poor prognostic factor for NPC (Tian et al., 2013), with a worse prognosis than lung and bone metastases (Hui et al., 2004). Recently, the presence of diffuse liver disease (steatosis, viral hepatitis, cirrhosis, and hepatic fibrosis) had been found to be protective against liver metastases in patients with solid tumors (Monelli et al., 2021), and a cohort study showed that fatty liver was a protective factor against hepatic metastases from breast cancer (Wu et al., 2017), and these findings sparked our interest.

Hepatitis B virus (HBV) infection was one of the major pathologic factors leading to chronic liver injury, liver-related immune changes and cirrhosis. Although most people were able to spontaneously clear the virus within six months, about 5 %−10 % of HBV-infected individuals developed chronic infection (Khanizadeh et al., 2019). At present, studies on NPC with liver metastasis were very limited, and the relationship between the incidence of liver metastasis and HBV infection status in NPC patients had not been studied. Therefore, this study recruited 967 patients with NPC diagnosed by pathology at Xiangya Cancer Hospital of Central South University from May 2017 to December 2022, quantified HBV-DNA, and further investigated the relationship between HBV-DNA and liver metastasis.

## 2 Patients and methods

### 2.1 Study population and criteria

This study selected 1047 patients with NPC diagnosed by histopathology from Hunan Province and its neighboring regions, including Jiangxi, Guizhou, Hubei and other places, from May 2017 to December 2022 for a cross-sectional study. The clinical stage of the patient was determined according to the 8th edition TNM staging system of the International Union for Cancer Control/American Joint Committee on Cancer (Amin et al., 2017). Exclusion criteria included: patients with missing clinical data, patients with a history of cancer or primary tumors, patients with severe and/or uncontrollable infections, pregnant or lactating female patients, and patients receiving antiviral treatment. A total of 976 NPC patients were finally included in this study. Among them, 530 cases with serological positivity of HBV surface antigen (HBsAg) for more than 6 months and/or positive HBV deoxyribonucleic acid (HBV-DNA) were included in the HBV-positive group, and the remaining 446 cases were in the HBV-negative group. Liver metastases were primarily confirmed by pathological or cytological examination, or were determined by the consensus of at least two experienced senior physicians based on imaging examinations and other laboratory tests. The study was approved by the Clinical Research Ethics Committee of Xiangya Cancer Hospital, Central South University, and was conducted in accordance with the Declaration of Helsinki (KYJJ-2023–156).

### 2.2 Detection of HBV viral load and clinically relevant liver parameters

EDTA-K_2_ anticoagulated peripheral blood of the subjects was collected, HBV-DNA and EBV-DNA were detected in accordance with the standard operating procedures of Shengxiang HBV Nucleic Acid Quantitative Detection Kit and EBV Nucleic Acid Quantitative Detection Kit (Changsha, China). Liver function biochemical parameters, including total protein (TP), albumin (ALB), alanine aminotransferase (ALT), aspartate aminotransferase (AST), total bilirubin (TBIL), direct bilirubin (DBIL), and indirect bilirubin (IBIL), were measured by Beckman Coulter AU5800 automatic biochemical analyzer. HBV serological markers were determined using Wantai Caris 200 automatic chemiluminescence immunoassay system (Beijing, China) with matched reagents. Meanwhile, studies had shown that for patients with HBV DNA≥2000 IU/mL, further antiviral treatment was required (Kaya et al., 2021), the HBV-positive group was further classified into the low HBV viral load group (HBV DNA <2000 IU/mL) and high HBV viral load group (HBV DNA ≥2000 IU/mL) to further compare the liver metastasis rate between the two groups.

### 2.3 Statistical analysis

All statistical analyses were performed using SPSS version 27.0 (IBM Corp, Armonk, NY). Continuous variables were expressed as mean (SD) ± standard deviation or median (Q1, Q3), and categorical variables were expressed as counts (%). Student's *t*-test was used for two independent normally distributed samples. The logarithmic EBV-DNA copy numbers were compared using the Mann-Whitney test. Categorical variables were presented as frequencies and percentages, and intergroup differences were analyzed using the chi-square (χ²) test or Fisher's exact test when expected counts were less than 5. Multivariate regression analysis was used to further analyze the risk factors of liver metastasis. Two-sided *p* values less than 0.05 were considered significant.

## 3 Results

**Fig. 1:**
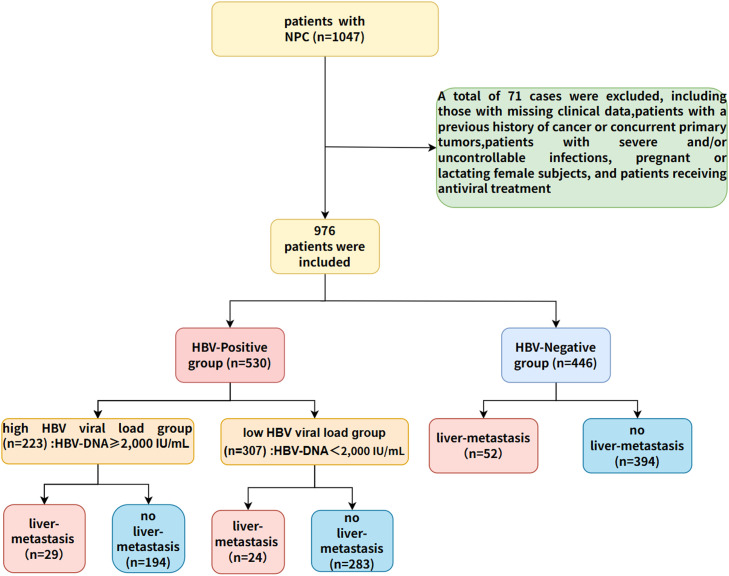
Profile of the study.

To explore the clinical significance of HBV infection in NPC, a total of 976 NPC patients were enrolled in this study. Based on serological results, the cohort was categorized into an HBV-negative group (*n* = 446) and an HBV-positive group (*n* = 530) (Fig. 1). As shown in Table 1, there were no significant differences in age, gender, chemoradiotherapy, EBV and liver metastasis rates across various stages between the HBV-negative and HBV-positive groups.

**Table 1:** Basic characteristics and liver metastasis of HBV negative group and HBV positive group.

	HBV-positive group *n* = 530	HBV-negative group *n* = 446	*p value*
Age (years)	48.44±9.93	49.87±9.78	0.783
Gender M/F	412/118	326/120	0.093(χ^2^=2.83)
Chemoradiotherapy (%)	513/530(97 %)	434/446(97 %)	0.636(χ^2^=0.225)
EBV [lg(copies/mL)]	2.60(2.60–2.97)	2.60(2.60–2.93)	0.454
Liver-Metastases ( %)	53/ 530(10.00 %)	52 /446(11.66 %)	0.405(χ^2^=0.694)
stage I-II	1/52(1.92 %)	0/52(0)	1.000(Fisher's)
stage III	4/78(5.13 %)	4/46(8.70 %)	0.454(χ² =0.223)
stage IV	48/400(12.00 %)	48/348(14.36 %)	0.492(χ² =0.473)

Further, subjects in the HBV-positive group were stratified into a high HBV viral load group (*n* = 223) and a low HBV viral load group (*n* = 307). As shown in Table 2, there were no significant differences in age, gender, TP, ALB, TBIL, DBIL, IBIL, chemoradiotherapy, TNM stage, and EBV load between the low HBV viral load group and the high HBV viral load group. The low HBV viral load group (*n* = 307) consisted predominantly of HBsAg-positive patients (96 %), with the remaining small proportion (4 %) of HBsAg-negative patients potentially representing window period or occult infection states (Raimondo et al., 2019). The high viral load group exhibited significantly higher levels of ALT and AST, as well as higher positive rates of HBsAg and HBeAg, compared to the low viral load group (*p* < 0.05). The liver metastasis rate of the low HBV viral load group (24/283, 7.82 %) was lower than that of the high HBV viral load group (29/194, 13.00 %) (*p* = 0.049) (Table 2). Meanwhile, combined with factors such as HBV-DNA high replication, chemoradiotherapy age, gender, TNM stage, and EBV, multiple regression analysis was conducted on liver metastasis in patients with HBV-positive NPC. The results showed a high HBV-DNA viral load (OR:12.661; 95 %CI 1.025–6.907, *p* = 0.044) was a risk factor for liver metastasis in NPC patients with HBV-positive.

**Table 2:** Basic characteristics and liver metastasis of low HBV viral load group and high HBV viral load group.

	low HBV viral load group *n* = 307	high HBV viral load group *n* = 223	*p value*
Age (years)	48.14±9.70	48.84±10.24	0.576
Gender M/F	237/70	175/80	0.022(χ^2^=5.23)
TP (g/L)	71.40(66.95–75.25)	72.40(67.75–75.60)	0.132
ALB (g/L)	42.85(39.70–45.00)	42.80(40.20–45.00)	0.977
ALT (U/L)	25.30(16.70–36.40)	31.85(21.85–48.58)	<0.001
AST (U/L)	25.70(21.50–32.00)	30.40(23.65–39.70)	<0.001
TBIL (μmol/L)	12.98(10.38–16.84)	13.38(10.40–17.97)	0.266
DBIL (μmol/L)	3.90(2.87–5.45)	3.96(2.76–5.34)	0.899
IBIL (μmol/L)	9.16(7.21–11.43)	9.50(7.07–12.72)	0.200
HBsAg positive	96.09 %	100.00 %	0.007(χ^2^=7.24)
HBeAg positive	0.98 %	8.97 %	<0.001(Fisher's)
HBeAb positive	95.44 %	91.93 %	0.136(χ²=2.22)
HBcAb positive	99.35 %	100.00 %	0.624(Fisher's)
Chemoradiotherapy	299/8(97.39 %)	214/9(95.96)	0.356(χ^2^=0.851)
Liver metastases	24/283(7.82 %)	29/194(13.00 %)	0.049(χ^2^=22.3)
EBV [lg(copies/mL)]	2.60(2.60–3.04)	2.60(2.60–2.99)	0.317
Tumor stage I-II/III-IV	28/279	24/199	0.530(χ^2^=0.393)

Seropositivity for HBV markers was defined as a value exceeding the upper limit of the manufacturer's reference range. The specific cut-off values were as follows: HBsAg, 0.20 IU/mL; HBeAg, 0.500 PEIU/mL; HBeAb, 0.20 PEIU/mL; HBcAb, 0.90 PEIU/mL.

## 4 Discussion

**Fig. 2:**
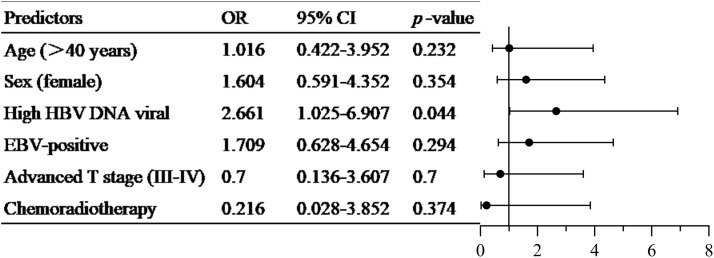
Multivariate regression analysis chart of high-risk factors related to liver metastasis in patients with HBV-positive NPC.

It was well known that HBV infection affected about 296 million people worldwide and was the leading cause of liver cirrhosis and liver cancer (Hsu et al., 2023). Autopsy reports of 5092 patients with malignant tumors found that 28.6 % of healthy liver patients developed liver metastasis, while only 4.5 % of cirrhotic patients developed liver metastasis (Lieber, 1957), suggesting that HBV infection might be related to malignant tumor liver metastasis. This study found that the liver metastasis rate of HBV-negative patients was 11.66 %, slightly higher than that of HBV-positive patients, but it was not statistically significant. In pancreatic cancer, HBsAg positive patients had a higher risk of developing liver metastases than HBsAg negative patients (Wei et al., 2013). However, chronic HBV infection found in colorectal cancer might reduce the risk of liver metastasis (Huo et al., 2018; Jiaming et al., 2020). These two different conclusions about the relationship between HBV infection and liver metastasis of malignant tumors aroused our interest. Whether the 'protection' or 'induction' of patients with HBV infection against liver metastasis of malignant tumors was related to HBV DNA levels? Based on current clinical guidelines and cutting-edge clinical trials, we further stratified the HBV-positive cohort into low and high viral load groups using a cut-off value of 2000 IU/mL (EASL 2017 2017; Shao et al., 2025; Tseng et al., 2019). Our analysis revealed that the liver metastasis rate of low HBV viral load group (7.28 %) was significantly lower than that of High HBV viral load group (13.00 %). The liver had a certain number of innate immune cells, especially lymphocytes including natural killer cells (Doherty et al., 1999) and several cells with phagocytosis and antigenic presentation properties, including Kupffer cells and dendritic cells, which played an important role in the local innate immunity of the liver (McDonald et al., 2009).HBV replication could activate cytotoxic lymphocytes (CTLS) and the specific lytic pathway of Kupffer cell damage (Budhu et al., 2006). Activated Kupffer cells could phagocytose circulating tumor cells, thereby inhibiting the metastasis of cancer cells to the liver to a certain extent (Matsumura et al., 2014). CTL cleared HBV in the liver by inducing Th1 type inflammatory factors (Wieland et al., 2000).When HBV replication was inhibited, these inflammatory factors induced activation of CD4+ T cells, NK, NKT cells, and toll-like receptors in the liver (Isogawa et al., 2005; Kakimi et al., 2000). In addition, HBV replication could promote the production of tumor necrosis factor a (TNF-α) by residual immune cells and hepatocytes in the liver (González-Amaro et al., 1994; Lara-Pezzi et al., 1998), which had certain anti-tumor effects. The liver microenvironment of HBV-positive metastatic HCC patients was found to have profound changes in the expression profile of more than 30% of genes related to inflammation and/or immune response function (Budhu et al., 2006). In conclusion, liver-related immune activation induced by HBV infection might partially explain the lower risk of liver metastasis in NPC patients in the low HBV viral load group. Studies had shown that maintaining relatively low levels of HBV DNA had a strong protective effect on the overall and long-term recurrence-free survival of HBV-associated HCC patients (An et al., 2010), which supported our conclusion to a certain extent. During HBV infection, specific T cells were enriched in the infected liver (Moriyama et al., 1990; Thimme et al., 2003), which activated the local immune microenvironment of the liver to a certain extent. However, the frequency and function of intrahepatic and peripheral HBV-specific T lymphocytes in regulating these processes was inversely proportional to the level of circulating HBV-DNA (Penna et al., 1996; Webster et al., 2004), and these diminished T cell responses were combined with persistently high levels of viruses and viral antigens. It leaded to progressive depletion and dysfunction of HBV-specific T cells (Wherry et al., 2003; Zhou et al., 2004), and further leaded to local immune dysfunction of the liver, which was consistent with the conclusion that the rate of liver metastasis was higher in NPC patients in the high HBV viral load group. Meanwhile, multivariate regression analysis was conducted in patients with HBV-positive NPC in combination with multiple factors such as high HBV-DNA replication, radiotherapy and chemotherapy, age, gender, TNM stage and EBV. The results indicated that high HBV viral load (OR: 2.661; 95 %CI 1.025–6.907) was a risk factor for HBV-positive NPC patients (Fig. 2). Tips for HBV with high viral load in NPC patients, should be timely for further treatment, and guard against risk of liver metastasis. More and more evidence indicated that HBV genotypes exhibit different disease manifestations and treatment responses (Zhang et al., 2021), which also leaded us to consider whether HBV genotypes were related to liver metastasis in malignant tumors. Further integration of genotype data for comprehensive analysis was needed.

## 5 Conclusion

Our study showed that HBV infection status in NPC patients was not significantly associated with liver metastasis; however, high HBV-DNA viral load was associated with an increased risk of liver metastasis in the HBV-infected population. In clinical practice, HBV-DNA should be actively monitored for NPC patients carrying HBV. For some patients with active viral replication, the risk of liver metastasis should be vigilant, which is conducive to improving the prognosis of NPC patients.

## Funding

This authors’ work was supported by the Hunan Cancer Hospital 2023 Sailing Youth Fund (QH2023008)

## Ethical approval

The study was approved by the Clinical Research Ethics Committee of Xiangya Cancer Hospital, Central South University, and was conducted in accordance with the Declaration of Helsinki (KYJJ-2023–156).

## Data availability

The datasets generated during and/or analysed during the current study are available from the corresponding author on reasonable request.

## Consent to participate

Informed consent was obtained from all individual participants included in the study.

## Author statement

We declare that this manuscript is original, has not been published before and is not currently being considered for publication elsewhere.

We confirm that the manuscript has been read and approved by all named authors and that there are no other persons who satisfied the criteria for authorship but are not listed. We further confirm that the order of authors listed in the manuscript has been approved by all of us.

We understand that the Corresponding Author Yizhi Peng is the sole contact for the Editorial process. She is responsible for communicating with the other authors about progress, submissions of revisions and final approval of proofs.

## CRediT authorship contribution statement

**Xiaofan Wang:** Writing – original draft, Methodology, Conceptualization. **Ying Bao:** Software, Methodology, Data curation. **Sheng Yin:** Writing – original draft, Methodology. **Yizhi Peng:** Writing – review & editing, Methodology, Funding acquisition, Data curation, Conceptualization.

## Declaration of competing interest

The authors declare no conflict of interest.

## Appendix Supplementary materials

Image, application 1
