# Supplementary material for: Association between high HBV-DNA viral load and liver metastasis risk in patients with nasopharyngeal carcinoma
Source: Virus Res. 2025 Oct 25;361:199651. doi: 10.1016/j.virusres.2025.199651 (PMC12607084; doi:10.1016/j.virusres.2025.199651)
Supplement: Supplementary file 1 [file mmc1.pdf]

**Subject**: **Correction Request for the First Affiliation** in the Manuscript "Association Between High HBV-DNA Viral Load and Liver Metastasis Risk in Patients with Nasopharyngeal Carcinoma" (**Manuscript ID: VIRUS-D-25-00099**)

To the Editorial Office of ***Virus Research***,

Dear Editors,

We are the authors of the manuscript titled "Association Between High HBV-DNA Viral Load and Liver Metastasis Risk in Patients with Nasopharyngeal Carcinoma" (Manuscript ID: VIRUS-D-25-00099), currently under consideration at your journal.

We are writing to respectfully request a change to the first author's affiliation for this manuscript.

Reason for Change:
Upon submission, we misunderstood the institutional affiliation designation process and incorrectly assumed that the corresponding author's institution would be automatically listed as the first affiliation. We have since realized this is not the case according to your journal's specific guidelines. Therefore, we wish to correct the first primary affiliation to accurately reflect the institution where the research was primarily conducted and ensure proper attribution.

Requested Change:
We hereby request that the first affiliation be changed from:
" Department of Clinical Laboratory, Taizhou Central Hospital (Taizhou University Hospital), Taizhou, Zhejiang Province, 318000, China "
to
" Department of Clinical Laboratory, Hunan Cancer Hospital/the Affiliated Cancer Hospital of Xiangya School of Medicine, Central South University, Changsha Province, 410031, China ".

We confirm that this change pertains solely to the modification of the first affiliation. The authorship order, the corresponding author designation, and all academic content of the manuscript remain entirely unchanged.

This request has been discussed and unanimously approved by all contributing authors. We sincerely apologize for any inconvenience this necessary correction may cause. Thank you very much for your understanding and assistance.

Sincerely,

Signatures of All Authors:

1. First Author:

Name (Printed): __Xiaofan Wang________

Signature: 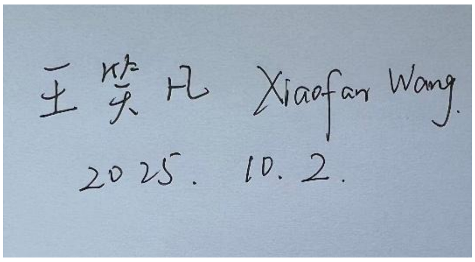


Affiliation: [Department of Clinical Laboratory, Taizhou Central Hospital (Taizhou University Hospital), Taizhou, Zhejiang Province, 318000, China]

2. Corresponding Author:

Name (Printed): __Yizhi Peng________

Signature: 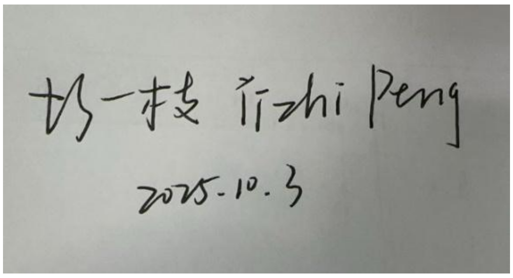

Affiliation: [Department of Clinical Laboratory, Hunan Cancer Hospital/the Affiliated Cancer Hospital of Xiangya School of Medicine, Central South University, Changsha Province, 410031, China] **First Affiliation**

3. Author:

Name (Printed): __ Ying Bao ___________

Signature: 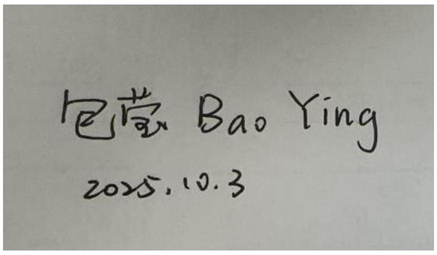


Affiliation: [Department of Clinical Laboratory, Hunan Cancer Hospital/the Affiliated Cancer Hospital of Xiangya School of Medicine, Central South University, Changsha Province, 410031, China]

4. Author:

Name (Printed): _Sheng Yin_____________

Signature: 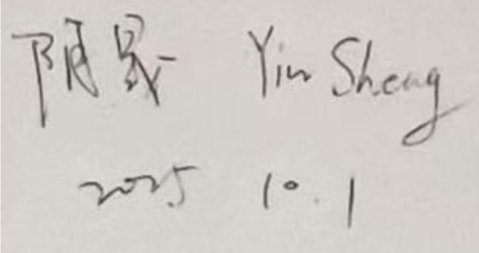

Affiliation: [Department of Clinical Diagnosis, Laboratory of Beijing Tiantan Hospital and Capital Medical University, No.119, South Fourth Ring West Road, Fengtai District, Beijing, 100070, China]
